# Genetic variants of MCP-1 and CCR2 genes and IgA nephropathy risk

**DOI:** 10.18632/oncotarget.12847

**Published:** 2016-10-24

**Authors:** Jie Gao, Xinghan Liu, Linting Wei, Dan Niu, Jiali Wei, Li Wang, Heng Ge, Meng Wang, Qiaoling Yu, Tianbo Jin, Tian Tian, Zhijun Dai, Rongguo Fu

**Affiliations:** ^1^ Department of Nephrology, Second Affiliated Hospital of Xi'an Jiaotong University, Xi'an 710004, China; ^2^ Department of Oncology, Second Affiliated Hospital of Xi'an Jiaotong University, Xi'an 710004, China; ^3^ Department of Nephrology, First Affiliated Hospital of Xi'an Jiaotong University, Xi'an 710061, China; ^4^ Department of Nephrology, Hainan general hospital, Haikou 570311, China; ^5^ Department of Pathology, Second Affiliated Hospital of Xi'an Jiaotong University, Xi'an 710004, China; ^6^ National Engineering Research Center for Miniaturized Detection Systems, School of Life Sciences, Northwest University, Xi'an 710069, China

**Keywords:** monocyte chemoattractant protein-1, IgA nephropathy, polymorphism, risk, case-control study

## Abstract

Monocyte chemoattractant protein-1 (MCP-1) and its receptor CCR2 stimulate inflammation response by activating and recruiting monocytes/macrophages. MCP-1 and CCR2 polymorphisms were reported to be associated with various diseases. To explore the relationship between MCP-1 and CCR2 polymorphisms and IgA nephropathy (IgAN), we conducted this case-control study by enrolling 351 IgAN patients and 310 health controls. Odds ratios (ORs) and 95% confidence intervals (CIs) were calculated to evaluate potential associations of MCP-1 and CCR2 polymorphisms with susceptibility and clinical parameters of IgAN. No statistical differences between IgAN group and the control group in the MCP-1 -2518 and CCR2 +190 genotypic groups were observed (*P* > 0.05). Individuals with MCP-1 -2518 GG genotypes had a higher blood pressure (GG vs. AA+AG: OR = 1.79, 95% CI = 1.07-2.99, *P* = 0.026) and Lee's grade (GG vs. AA+AG: OR = 2.05, 95% CI = 1.19-3.54, *P* = 0.009; GG vs. AA: OR = 2.24, 95% CI = 1.19-4.20, *P* = 0.01), compared with patients with AA/AG genotypes. A significant association between CCR2 +190 polymorphism and Lee's grades was observed (GA+AA vs. GG: OR = 2.66, 95% CI = 1.63-4.35, *P* < 0.001; GA vs. AA+GG: OR = 2.27, 95% CI = 1.39-3.70, *P* = 0.001). Our results indicated that MCP-1 and CCR2 polymorphisms may influence the progression of IgAN, but not increase/decrease its susceptibility.

## INTRODUCTION

IgA nephropathy (IgAN), which is also called Berger's disease, is the most frequent glomerulonephritis characterized by the deposition of IgA1-based immune complexes in mesangium [1, 2]. Although some new biomarkers exist, renal biopsy is the gold standard for the diagnosis of IgAN [3]. Proteinuria and hematuria were the main clinical manifestations for IgAN and increasing proteinuria usually associated with worse clinicopathologic features in IgAN patients [4, 5]. IgAN patients with mild symptoms often coincide with an upper respiratory tract infection at initial, but 50% of these patients will develop into end-stage renal disease within the next 20 years [6]. Air pollution, low socioeconomic status, and gene are the risk factors for IgAN [7]. In a test including 148 healthy female twins, the heritability of serum undergalactosylated IgA1 and IgA levels were found to be 80% and 46%, respectively, which indicates the importance of genetic factors in pathogenesis of IgAN [8]. Genome-wide association studies also reported polymorphisms in gene were strongly correlated with IgAN prevalence and prognosis, but the associations and the magnitude are needed to confirm [9, 10].

Monocyte chemoattractant protein-1 (MCP-1), which is also known as C-C chemokine ligand 2 (CCL2), promotes the recruitment of monocytes, macrophages, and other inflammatory cells to inflammation site by interaction with its receptor CCR2 [11]. In MCP-1-intact mice models, MCP-1 was mainly localized in cortical tubules and induced tubular injury by recruiting macrophages [12]. MCP-1 plays a vital role in the progression of renal diseases. Liu et al. found human serum albumin could stimulate proximal tubular epithelial HK-2 cells to produce MCP-1, and the effect was obvious when its concentration was more than 2g/L [13]. Sun et al. suggested a positive association between MCP-1 in tubulointerstitial and 24h urinary protein excretion, which indicated high urinary protein in IgAN patients may stimulate the expression of MCP-1 and further promote and activate monocytes/macrophages, finally leading to renal injury and interstitial fibrosis [14]. CCR2 is the specific receptor of MCP-1, and the MCP-1/CCR2 signal axis was involved in the transmission of cell information and cell migration, which could enhance cell proliferation and migration ability of renal cell carcinoma by autocrine [15]. High expression of MCP-1 and CCR2 were significantly associated with shortened survival time and increased risk of recurrence in patients carrying non-metastatic clear-cell renal cell carcinoma and CCL2/CCR2 signature had a negative effect on overall survival and recurrence-free survival [16]. In view of the correlation between MCP-1 and CCR2 and their synergistic effect in biological function, there were a large number of researches focused on their SNPs together [11, 17–19], in order to find a meaningful haplotype combination. A previous study by Mandal et al. stated that MCP-1 -2518A/G and CCR2 +190G/A polymorphisms had a synergistic relationship [11].

Recent articles have reported the association between MCP-1 and CCR2 gene polymorphisms and various diseases. A meta-analysis indicated MCP-1 -2518A/G polymorphism was significantly associated with lupus nephritis occurrence in Caucasians [20]. MCP-1 -2518AA genotype was a risk for diabetes mellitus patients developing into diabetic nephropathy [21]. CCR2 +190G/A polymorphism had no association with diabetic nephropathy in Japanese [22]. Only one article evaluated the association between MCP-1 -2518A/G polymorphism and primary glomerulonephritis, but the authors failed to find any correlation in the Polish population [23].

Till now, few studies had investigated the association of MCP-1 -2518A/G and CCR2 +190G/A polymorphisms with IgAN susceptibility, but the results were inconsistent. Therefore, we conducted this case-control study to evaluate the association in the Chinese population.

## RESULTS

### Characteristics of the participants

We recruited 351 IgAN patients as the case group and 310 health persons as the control group. The characteristics of the two groups were listed in Table 1. The average age in cases and controls had no statistical difference (32±11.9 vs. 35±12.6, *P* = 0.45). 65% of the patients and 60% of the health were male (*P* = 0.16). The frequencies of patients that had a 24h urine protein <3.5g/24h, blood pressure <140/90mmHg, and I+II+III Lee's classifications were 77.5%, 55.3%, and 74.1%, respectively. The average of serum creatinine, blood urea nitrogen, serum albumin, IgA, and C3 levels were 159.5±146.0 umol/L, 8.2±5.9 mmol/L, 34.01±7.98 g/L, 2.76±1.72 g/L, and 1.06±0.26 g/L, respectively. The genotypes of MCP-1 -2518 and CCR2 +190 polymorphisms in the control group were in Hardy-Weinberg equilibrium (*P* = 0.34 and 0.95, respectively).

**Table 1:** Basic characteristics of the subjects

Total subjects (n=661)	IgAN	Control	*P*
Number of subjects (n)	351	310	
Male/Female	229/122	186/124	0.16^a^
Age (mean±SD)	32±11.9	35±12.6	0.45^b^
24h urine protein (g/24h)			
<3.5	272 (77.5%)		
≥3.5	79 (22.5%)		
blood pressure (mmHg)			
<140/90	194 (55.3%)		
≥140/90	157 (44.7%)		
Lee's classification			
I+II+III	260 (74.1%)		
IV+V	91 (25.9%)		
Serum Cr (μmol/L)	159.5±146.0		
BUN (mmol/L)	8.2±5.9		
Serum ALB (g/L)	34.01±7.98		
Serum IgA (g/L)	2.76±1.72		
Serum C3 (g/L)	1.06±0.26		

a *P* values was calculated from two-sided chi-square test;

b *P* values were calculated by Student *t* tests.

### Associations between genotype frequencies and IgAN risk

As shown in Table 2, MCP-1-2518 AG genotype decreased the IgAN risk to 0.90 and GG genotype increased the IgAN risk to 1.20 compared with AA genotype, but there was no statistical difference (AG vs. AA: OR = 0.90, 95% CI = 0.64-1.26, *P* = 0.53; GG vs. AA: OR = 1.20, 95% CI = 0.78-1.85, *P* = 0.40). The *P* values were also not significant in other comparisons (Dominant model: OR = 0.98, 95% CI = 0.72-1.35, *P* = 0.91; Recessive model: OR = 1.28, 95% CI = 0.86-1.88, *P* = 0.22; Overdominant model: OR = 0.84, 95% CI = 0.62-1.15, *P* = 0.27; Log-additive model: OR = 1.06, 95% CI = 0.86-1.31, *P* = 0.56). The GG, GA and AA genotype frequencies of CCR2 +190 polymorphisms in cases were 51.8%, 40.5%, and 7.8%, and in controls were 58.4%, 34.2%, and 7.4%, respectively. We failed to find any significant association between CCR2 +190 polymorphism and the susceptibility to IgAN in any comparison (Table 3).

**Table 2:** Relationships between MCP-1 -2518 polymorphism and IgA nephropathy risk

Model	Genotype	Control	Case	OR (95% CI)	*P*-value
Codominant	A/A	115 (37.1%)	131 (37.5%)	1.00	
	A/G	141 (45.5%)	144 (41.3%)	0.90 (0.64-1.26)	0.53
	G/G	54 (17.4%)	74 (21.2%)	1.20 (0.78-1.85)	0.40
Dominant	A/A	115 (37.1%)	131 (37.5%)	1.00	0.91
	A/G-G/G	195 (62.9%)	218 (62.5%)	0.98 (0.72-1.35)	
Recessive	A/A-A/G	256 (82.6%)	275 (78.8%)	1.00	0.22
	G/G	54 (17.4%)	74 (21.2%)	1.28 (0.86-1.88)	
Overdominant	A/A-G/G	169 (54.5%)	205 (58.7%)	1.00	0.27
	A/G	141 (45.5%)	144 (41.3%)	0.84 (0.62-1.15)	
Log-additive	---	---	---	1.06 (0.86-1.31)	0.56

OR: odds ratio, 95% CI: 95% confidence interval.

**Table 3:** Relationships between CCR2 +190 polymorphism and IgA nephropathy risk

Model	Genotype	Control	Case	OR (95% CI)	*P*-value
Codominant	G/G	160 (51.8%)	205 (58.4%)	1.00	
	G/A	125 (40.5%)	120 (34.2%)	0.75 (0.54-1.04)	0.08
	A/A	24 (7.8%)	26 (7.4%)	0.85 (0.47-1.53)	0.58
Dominant	G/G	160 (51.8%)	205 (58.4%)	1.00	0.09
	G/A-A/A	149 (48.2%)	146 (41.6%)	0.76 (0.56-1.04)	
Recessive	G/G-G/A	285 (92.2%)	325 (92.6%)	1.00	0.86
	A/A	24 (7.8%)	26 (7.4%)	0.95 (0.53-1.69)	
Overdominant	G/G-A/A	184 (59.5%)	231 (65.8%)	1.00	0.10
	G/A	125 (40.5%)	120 (34.2%)	0.76 (0.56-1.05)	
Log-additive	---	---	---	0.84 (0.66-1.07)	0.16

OR: odds ratio, 95% CI: 95% confidence interval.

### Associations between genotype frequencies and IgAN clinical parameters

To figure out if the two polymorphisms affect the progression of IgAN or have the gender difference, we investigated the correlation between the two polymorphisms and a series of indexes. According to gender, 24h urinary protein (3.5g), blood pressure (140/90mmHg) and Lee's grades (III), IgAN patients were divided into two groups. MCP-1 -2518 genotypes distribution had no difference between different genders or 24h urinary protein of patients (Table 4). Compared with individuals with -2518 AA/AG genotypes, patients with GG genotypes had a higher blood pressure (Table 4, GG vs. AA+AG: OR = 1.79, 95% CI = 1.07-2.99, *P* = 0.026; GG vs. AA: OR = 1.85, 95% CI = 1.04-3.28, *P* = 0.035) and Lee's grade (Table 4, GG vs. AA+AG: OR = 2.05, 95% CI = 1.19-3.54, *P* = 0.009; GG vs. AA: OR = 2.24, 95% CI = 1.19-4.20, *P* = 0.01). In male patients, CCR2 +190 GG genotype accounts for 57.6%, GA+AA genotypes account for 57.6% and 35.4%, which were similar to the proportion in female patients (Table 4, GA+AA vs. GG: OR = 1.09, 95% CI = 0.70-1.71, *P* = 0.69). Under all the comparison models, we did not find the relationship of CCR2 +190 polymorphism with 24h urinary protein or blood pressure (Table 4). However, a significant association between CCR2 +190 polymorphism and Lee's grades was observed (Table 4, GA+AA vs. GG: OR = 2.66, 95% CI = 1.63-4.35, *P* < 0.001; GA vs. AA+GG: OR = 2.27, 95% CI = 1.39-3.70, *P* = 0.001).

**Table 4:** The association between MCP-1 -2518 and CCR2 +190 polymorphisms and clinical characteristics in IgAN patients

MCP-1 -2518	AA	AB+BB	*P*	OR (95%CI)	AA+AB	BB	*P*	OR (95%CI)	AA+BB	AB	*P*	OR (95%CI)
Gender												
Female	45	50	0.95	0.98 (0.60-1.62)	95	26	0.92	0.97 (0.57-1.67)	71	50	0.99	1.00 (0.64-1.56)
Male	86	94			180	48			134	94		
24h urine protein												
<3.5	99	171	0.54	0.85 (0.51-1.42)	207	63	0.07	0.53 (0.27-1.07)	162	108	0.38	1.26 (0.76-2.08)
≥3.5	32	47			68	11			43	36		
Blood pressure												
<140/90	77	116	0.26	1.29 (0.83-2.00)	160	33	**0.026**	1.79 (1.07-2.99)	110	83	0.46	0.85 (0.55-1.31)
≥140/90	53	103			114	42			95	61		
Lee's grade												
I-III	103	155	0.12	1.49 (0.90-2.49)	212	46	**0.009**	2.05 (1.19-3.54)	149	109	0.53	0.85 (0.53-1.39)
IV-V	28	63			63	28			56	35		
**CCR2 +190**												
Gender												
Female	73	49	0.69	1.09 (0.70-1.71)	112	10	0.68	0.84 (0.37-1.92)	83	39	0.52	1.16 (0.73-1.86)
Male	132	97			213	16			148	81		
24h urine protein												
<3.5	157	115	0.63	0.88 (0.53-1.47)	250	22	0.37	0.61 (0.20-1.81)	179	93	0.99	1.00 (0.59-1.70)
≥3.5	48	31			75	4			52	27		
Blood pressure												
<140/90	119	75	0.22	1.31 (0.86-2.01)	183	11	0.17	1.76 (0.78-3.94)	130	64	0.60	1.13 (0.72-1.75)
≥140/90	86	71			142	15			101	56		
Lee's grade												
I-III	168	92	**<0.001**	2.66 (1.63-4.35)	244	16	0.13	1.88 (0.82-4.31)	184	76	**0.001**	2.27 (1.39-3.70)
IV-V	37	54			81	10			47	44		

A: the major allele; B: the minor allele; OR: odds ratio; 95% CI: 95% confidence interval

### Associations between MCP-1-2518 and CCR2 +190 haplotypes and IgAN risk

To explore the interaction between the two polymorphisms, we further constructed the haplotype model. As shown in Table 5, comparing to A_-2518_G_+190_ haplotype, A_-2518_A_+190_ and G_-2518_A_+190_ haplotypes had no significant effect on IgAN risk (A_-2518_A_+190_ vs. A_-2518_G_+190_: OR = 0.80, 95% CI = 0.64-1.02, *P* = 0.07; G_-2518_A_+190_ vs. A_-2518_G_+190_: OR = 1.04, 95% CI= 0.75-1.46, *P* = 0.81).

**Table 5:** The associations between MCP-1 -2518 and CCR2 +190 haplotypes and IgAN risk

−2518	+190	Controls	Cases	*P*	OR (95%CI)
A	G	275	338		1
A	A	267	264	0.07	0.80 (0.64-1.02)
G	A	78	100	0.81	1.04 (0.75-1.46)

## DISCUSSION

Chemokines, with 8 to 12kD molecular weights, are divided into four subfamilies (CXC, CC, C, and CX3C) by their N-terminal cysteine-motifs [24]. MCP-1, belongs to the CC subfamily, has a strong chemotaxis and can activate the monocytes/macrophages, lead to the infiltration and activation of mononuclear cells, and the secretion of various inflammatory factors and fibrosis factors. An increased number of tubulointerstitial macrophages was significantly associated with a poor prognosis in IgAN patients [25]. Macrophages could activate nuclear factor-kappa B (NF-κB) and active protein-1 (AP-1), up-regulate the expression of multiple cytokines, chemokines and adhesion factors, such as tumor necrosis factor-9 and matrix metalloprotein-9, induce mesangial cell proliferation and matrix deposition, and further aggravate the kidney damage [26, 27]. NF-κB and MCP-1 increasingly expressed in glomeruli and interstitium was correlated with progression of tissue injury in IgAN [28]. In IgAN patients, the MCP-1 level was positively correlated with 24h urinary protein, renal tubular interstitial damage and serum creatinine, which indicated MCP-1 may be associated with IgAN progression [29]. IgA1 deposition, as the initiating factor in IgAN, can stimulate mesangial cells to produce MCP-1 [30], which eventually leaidng to the activation and accumulation of monocytes/macrophages and contributing to renal interstitial inflammation in kidney [31], Stangou et al. found that in IgAN patients, MCP-1 was positively associated with severe extracapillary proliferation (*P* = 0.001) and Th2 and Th17 cytokines were directly involved in renal pathology in IgAN through regulating the MCP-1 production [32]. In this study, we found MCP-1 -2518 polymorphism was related with IgAN patients' blood pressure and Lee's grades, except IgAN risk, what were consistent with the results of a previous study. Steinmetz et al. performed a case-control study with 207 IgAN patients and 140 controls, and revealed that patients with different MCP-1 -2518 genotypes had no difference in cumulative survival, median survival time or 5 year survival rate and MCP-1 -2518 polymorphism had no association with IgAN risk or clinical course [33]. However, in a study in Japan, the authors suggested that MCP-2518 AA genotype increased the risk of end stage renal disease and the progression of renal disease of IgAN patients compared with AG/GG genotype and individuals with AA genotype had a worse prognosis [34]. Different conclusions may result from ethnic difference, sample size or environmental factors.

CCR2, the receptor for MCP-1, play an important role in inflammatory disorders and some diseases [35, 36]. MCP-1/CCR2 signaling was involved in human crescentic glomerulonephrtitis and murine lupus nephritis [37, 38]. In a renal fibrosis model, the deletion of transient receptor potential vanilloid type 1 aggravated renal injury in salt-sensitive hypertension by enhancing MCP-1/CCR2 signaling-dependent inflammatory responses [39]. Blocking of CCR2 could improve the progressive fibrosis by decreasing macrophages in the diseased kidneys [40]. CCR2 gene is located in the region of chemokine receptor gene cluster and CCR2 is mainly produced by memory T cells, monocytes, dendritic cells, B cells and eosinophils. CCR2 +190 polymorphism is located at codon 64 of CCR2 gene with a single-nucleotide variation of G to A. Under the stimulation of inflammation, CCR2 could increase the expression of MCP-1. Our study firstly investigated the relationship of CCR2 +190 polymorphism with IgAN risk and clinical parameters. The association between CCR2 +190 polymorphism and Lee's grades was confirmed.

Compared with previous studies, we expand the sample size to 661 and performed the stratification analysis according to clinical characteristics in IgAN patients and the haplotype analysis to explore the interaction of the two polymorphisms. But several limitations exist in this study. Firstly, we only included 351 IgAN patients and 310 health controls, so the sample size may be small. Secondly, selection bias was inevitable. Thirdly, we only investigated the relationship in the Northwestern population, which is not on behalf of all areas.

In summary, we found an association between MCP-1 -2518 and IgAN patients' blood pressure and Lee's grades and an relationship between CCR2 +190 polymorphisms and Lee's grades, which suggested the two polymorphisms may affect the progression of IgAN. Therefore, more studies with larger sample size and different races are still needed to validate our study.

## MATERIALS AND METHODS

### Ethics statement

The study protocol was approved by the ethics committee of the Second Affiliated Hospital of Xi'an Jiaotong University. Written informed consent was obtained from all participants after a full explanation of the study. The experimental protocol was implemented in accordance with the approved guidelines.

### Subjects

351 patients, which were diagnosed as IgAN by renal biopsy and non-familial IgAN cases, were enrolled from Northwestern China at the First and Second Affiliated Hospital of Xi'an Jiaotong University from March 2009 to April 2014. 310 healthy subjects were recruited from routine healthy examinations in the same hospitals. All subjects were unrelated Chinese Han people living in Xi'an city or nearby. Patients would be excluded if they had comorbidities such as diabetes mellitus, lupus nephritis, and other secondary IgAN. Demographic and clinical details were collected, including age, gender, 24 hour urinenary protein, blood pressure, serum creatinine level (SCr), blood urea nitrogen (BUN), serum albumin level (ALB), serum cholesterol level (Cho), serum IgA level, serum C3 level, and histopathological grade (Lee's classification).

### DNA extraction and genotyping

Blood samples were collected in tubes containing ethylene diaminetetraacetic acid (EDTA) and stored at -80C after centrifugating at 1,500 rpm for 10 min. Genomic DNA from whole blood was extracted using the GoldMag DNA Purification Kit (GoldMag Co. Ltd, Xi'an City, China), and the purity and concentration was measured utilizing an ultraviolet spectrophotometer (Nanodrop, Thermo Scientific, Waltham, MA). The Sequenom MassARRAY Assay Design 3.0 software was used to design Multiplexed SNP MassEXTEND assay. SNP genotyping was performed by using Sequenom MassARRAY RS1000 according to the standard protocol. The primers used for MCP-1 -2518 and CCR2 +190 are listed in Table 6. Sequenom Typer 3.0 software was used for data analysis.

**Table 6:** Primers used for this study

SNP-ID	1st-PCRP	2nd-PCRP	UEP_SEQ
MCP-1-2518	ACGTTGGATGGAAG GTGAAGGGTATGAATC	ACGTTGGATGGGAG GGCATCTTTTCTTGAC	AAGTCTTCT GGAAAGTGA
CCR2 +190	ACGTTGGATGTGTC AGTCAAGCACTTCAGC	ACGTTGGATGTCTAC TCGCTGGTGTTCATC	GCAGTTTATT AAGATGAGGA

### Statistical analysis

SPSS 18.0 statistical package was used for all the data analysis (SPSS, Chicago, IL, USA). The SNP frequency in the controls was assessed for departure from Hardy–Weinberg Equilibrium (HWE) using an exact test. An χ^2^ test was conducted for calculating allele and genotype frequencies of cases and controls. Odds ratios (ORs) and 95% confidence intervals (CIs) were determined using unconditional logistic regression with adjustment for age and gender. Five genetic models (codominant, dominant, recessive, overdominant, and log-additive) were used to evaluate potential association of MCP-1 and CCR2 polymorphisms with risk and clinical parameters of IgAN. *P* < 0.05 was considered statistically significant and all statistical tests were two-sided.
